# HIPEC-Induced Acute Kidney Injury: A Retrospective Clinical Study and Preclinical Model

**DOI:** 10.1245/s10434-021-10376-5

**Published:** 2021-07-14

**Authors:** Lukas F. Liesenfeld, Benedikt Wagner, H. Christian Hillebrecht, Maik Brune, Christoph Eckert, Johannes Klose, Thomas Schmidt, Markus W. Büchler, Martin Schneider

**Affiliations:** 1 Department of General, Visceral and Transplantation Surgery, University Hospital Heidelberg, Heidelberg, Germany; 2 Department of Internal Medicine I and Clinical Chemistry, University Hospital Heidelberg, Heidelberg, Germany; 3 Department of Pathology, University Hospital Heidelberg, Heidelberg, Germany

## Abstract

**Background:**

Hyperthermic intraperitoneal chemotherapy (HIPEC) combined with cytoreductive surgery (CRS) is the treatment of choice for selected patients with peritoneal malignancies. HIPEC is accompanied by moderate-to-high patient morbidity, including acute kidney injury. The significance of nephrotoxic agents such as cisplatin versus hyperthermia in HIPEC-induced nephrotoxicity has not been defined yet.

**Patients and Methods:**

A total of 153 patients treated with HIPEC were divided into groups with (AKI+) and without (AKI−) kidney injury. Laboratory parameters and data concerning patient demographics, underlying disease, surgery, complications, and HIPEC were gathered to evaluate risk factors for HIPEC-induced AKI. A preclinical mouse model was applied to assess the significance of cisplatin and hyperthermia in HIPEC-induced AKI, as well as protective effects of the cytoprotective agent amifostine.

**Results:**

AKI occurred in 31.8% of patients undergoing HIPEC. Treatment with cisplatin-containing HIPEC regimens represented a major risk factor for HIPEC-related AKI (*p* < 0.001). Besides, angiotensin receptor blockers and increased preoperative creatinine and urea levels were independent risk factors for AKI after HIPEC. In a preclinical mouse model, intraperitoneal perfusion with cisplatin induced AKI, whereas hyperthermia alone, or in combination with cisplatin, did not induce or enhance renal injury. Amifostine failed to confer nephroprotective effects in a miniaturized HIPEC model.

**Conclusions:**

AKI is a frequent complication after HIPEC. The risk of renal injury is particularly high in patients treated with cisplatin-containing HIPEC regimens. Hyperthermic perfusion of the abdomen by itself does not seem to induce or aggravate HIPEC-induced renal injury.

**Supplementary Information:**

The online version contains supplementary material available at 10.1245/s10434-021-10376-5.

## Background

Cytoreductive surgery (CRS) combined with hyperthermic intraperitoneal chemotherapy (HIPEC) has become a standard treatment for patients with peritoneal carcinomatosis (PC) and pseudomyxoma peritonei (PMP).^1^ The peritoneum is a frequent metastatic site for tumors of colorectal, appendiceal, ovarian, gastric, or pancreatic origin, and the origin of peritoneal mesothelioma, a rare primary malignancy.^2^–^4^ Synchronous or metachronous peritoneal disease occurs in approximately 10% of colorectal cancer patients, and in 60% or 70% of ovarian cancer patients, and 14% or 50% of gastric cancer patients, respectively.^5^–^10^ Independent of primary tumor origin, peritoneal disease is associated with poor survival.^6^,^11^–^13^ According to a recent randomized controlled trial, cisplatin HIPEC improves survival of patients undergoing complete surgical cytoreduction of PC from ovarian cancer.^14^

Acute kidney injury (AKI) is a common complication of major abdominal surgery.^15^ In patients undergoing cytoreductive surgery and HIPEC, the prevalence of postoperative AKI varies widely between 1% and 48% (Table 1).^16^,^17^,^26^,^27^,^18^–^25^ Several risk factors for HIPEC-induced AKI have been identified, comprising age, obesity, intraoperative blood loss, baseline creatinine/glomerular filtration rate (GFR),preoperative albumin, and use of angiotensin-II receptor antagonists.^20^,^23^,^25^,^26^ Given its nephrotoxic properties in systemic treatment regimens, cisplatin is a likely contributor to HIPEC-induced AKI.^22^,^28^ Studies defining cisplatin as an independent risk factor for HIPEC-induced AKI are, however, inconsistent, and an alternative hypothesis holds that HIPEC-induced AKI is a consequence of relative hypovolemia, causing prerenal kidney damage due to hyperthermia-induced splanchnic vasodilatation.^16^,^18^,^24^,^29^,^30^ The aim of this study was, therefore, to analyze the prevalence and risk factors for AKI in patients undergoing HIPEC, and to evaluate the respective relevance of hyperthermia versus cytotoxic drugs such as cisplatin in the pathogenesis of HIPEC-induced AKI applying a preclinical mouse model.^31^

**Table 1:** Literature review of acute kidney injury after HIPEC

Author	Year	*N*	AKI (%)	Classification/cut-off	HIPEC regimens
Glehen et al.^16^	2003	216	1.3	> 3 × ULN (NCI CTC)	CPL (1 mg/kg, max. 80 mg), MMC (0.7 mg/kg, max. 60 mg), CPL (0.7 mg/kg) + (MMC 0.5 mg/kg)
Verwaal et al.^17^	2004	102	4.9	> 3 × ULN (NCI CTC)	MMC (25–40 mg/m^2^)
Kusamura et al.^18^	2007	247	5.7	> 3 × ULN (NCI CTC)	CPL (25 mg/m^2^/L) + MMC (3.3 mg/m^2^/L), CPL (43 mg/L) + DOX (15.25 mg/L)
Canda et al.^19^	2013	30	26.1	> 3 × ULN (NCI CTC)	CPL (75 mg/m^2^), MMC (10 mg/m^2^), CPL (75 mg/m^2^) + MMC (10 mg/m^2^)
Haslinger et al.^43^	2013	112	0.9	n/a	MMC (30 mg + 10 mg at 60 min)
Hakeam et al.^20^	2014	53	3.7	RIFLE	CPL (50 mg/m^2^) + DOX (15 mg/m^2^)
Arjona-Sanchez et al.^21^	2016	141	30.5	RIFLE	MMC (15 mg/m^2^), TAX (60 mg/m^2^)
Bouhadjari et al.^22^	2016	21	33	CreaCl < 30 ml/min	CPL (75 mg/m^2^) + MMC (20 mg/m^2^)
Sin et al.^23^	2017	47	40.4	> 3 × ULN (NCI CTC)	CPL (90 mg/kg)
Naffouje et al.^24^	2018	58	20.7	KDIGO	MMC (40 mg), CPL (45 mg/L) ± DOX (15 mg/L), MEL (50 mg/m^2^)
Ye et al.^25^	2018	99	11.1	RIFLE	5-FU (700–800 mg/m^2^) ± CPL (60 mg/m^2^)
Cata et al.^26^	2018	475	21.3	AKIN	CPL (n/a), OXA (n/a), MMC (n/a)
Angeles et al.^27^	2019	66	48	RIFLE	CPL (50–100 mg/m^2^)
Present study	2020	157	31.8	KDIGO	Multiple regimens (see Table 2)

*HIPEC* hyperthermic intraperitoneal chemotherapy, *ULN* upper limit normal, *NCI CTC* National Cancer Institute Common Terminology Criteria, *CreaCl* creatinine clearance, *RIFLE* risk, injury, failure, loss, and end-stage kidney disease, *AKIN* Acute Kidney Injury Network, *KDIGO* Kidney Disease Improving Global Outcome, *CPL* cisplatin, *MMC* mitomycin C, *DOX* doxorubicin, *TAX* taxol, *MEL* melphalan, *5-FU* 5-fluorouracil, *OXA* oxaliplatin, *n/a* not available

## Patients and Methods

### Study Design, Patient Population, and Data Source

This study was approved by the local ethical committee of the Medical Faculty Heidelberg. Between 2007 and 2016, 157 HIPEC treatments were performed in 153 patients at the Department of General, Visceral and Transplant Surgery of the University Hospital Heidelberg. Patients were divided into two groups according to the absence (AKI−) or presence (AKI+) of postoperative AKI. Data concerning patient demographics, laboratory results [creatinine, urea, white blood cell count (WBCC), C-reactive protein (CRP), lactate dehydrogenase (LDH), albumin, and Quick’s value], complications, and surgical and HIPEC details were extracted from the Health Management Information System, surgical, and anesthesiological reports.

### Cytoreductive Surgery, HIPEC, and EPIC

HIPEC was performed if patients’ peritoneal cancer index (PCI) did not exceed 20 and a completeness of cytoreduction score ≤ 1 could be achieved.^32^,^33^ All patients underwent exploratory laparotomy and CRS if macroscopically detectable peritoneal nodules were present.^34^ Closed-abdomen HIPEC was performed applying two inflow and outflow drains with attached thermal probes. For perfusion, a heart–lung machine was used with an inflow temperature of 41–43 °C. Perfusate volume varied according to abdominal cavity volume. Perfusion duration was 30 min for oxaliplatin-containing HIPEC and 90 min for all other HIPEC regimens. Eleven distinct HIPEC regimens were applied, three of which were followed by early postoperative intraperitoneal chemotherapy (EPIC). EPIC treatment was performed daily for 5 days postoperatively.

### Acute Kidney Injury

Renal function was assessed by blood parameters and urine output (UO) applying Kidney Disease Improving Global Outcomes (KDIGO) criteria (AKI stage I: increase in serum creatinine > 0.3 mg/dl within 48 h or UO < 0.5 ml/kg/h during 6 h; stage II: increase in creatinine 2–2.9 times baseline or UO < 0.5 ml/kg/h during 12 h; stage III: increase in serum creatinine > 4 mg/dl or 3 times baseline, or UO < 0.3 ml/kg/h during 24 h, or renal replacement therapy).^35^ Creatinine and urea were analyzed on postoperative days (POD) 0, 2, 4, 6, 8, and 10, and intra- and postoperative urine output were assessed until POD 2. Fluid balance was calculated on the day of surgery as well as on POD 1 and 2, respecting intravenous (i.v.) intake, oral intake and output (nasogastric tube), urine output, blood loss, and drains. Indications for renal replacement therapy were set by an interdisciplinary team. No renal protectants were routinely utilized in patients.

### Animals

White (albino) CD-1 IGS outbred mice [Crl:CD1 (ICR)] were procured from Charles River (Sulzfeld, Germany) and housed in a specified pathogen-free environment on a 12 h/12 h light/dark cycle with drink and feed ad libitum in accordance with institutional and federal animal welfare regulations. All experiments were approved by the responsible Karlsruhe Regional Council, Germany.

### Animal Model

Six-week-old animals had 150 µl of blood withdrawn via tail vein puncture. To avoid alteration of renal function due to blood withdrawal, 200 µl of sterile saline was substituted subcutaneously and animals recovered for 7 days. HIPEC was performed applying a closed circuit using a peristaltic pump and a water bath under isoflurane anesthesia, with saline 0.9% as perfusate solution.^31^ Cisplatin (75 mg/m^2^; prepared at the University Hospital Heidelberg pharmaceutical department) was added when the desired temperature was reached, and HIPEC performed for 90 min. HIPEC inflow temperature was 38 °C (normothermia) or 41–42 °C (hyperthermia). Perfusate volume was 2 L/m^2^. Amifostine (200 mg/kg; Selleck Chemicals LLC, Munich, Germany) was dissolved in sterile water (42 mg/ml) and administered subcutaneously (s.c.) 10–15 min prior to HIPEC. For postoperative analgesia, buprenorphine (0.1 mg/kg) was injected s.c. three times daily for 3 days. Three days after HIPEC, bilateral nephrectomy was performed, followed by blood withdrawal via cardiac puncture. Tissues were fixed in 4% paraformaldehyde (PFA) (Otto Fischar GmbH & Co. KG, Germany) and embedded in paraffin.

### Blood Samples

Creatinine, urea, and cystatin C plasma levels were analyzed in the accredited central laboratory of Heidelberg University Hospital on an ADVIA Chemistry XPT automated analyzer, using appropriate reagent kits (Siemens, cat. nos. 508029, 06860558, and 04851534, respectively) according to the manufacturer’s protocol.

### Histology

Paraffin sections were stained with periodic acid–Schiff (PAS) to detect nuclear pathologies, loss of tubule brush border, hyaline cast formation, tubular dilatation, and disruption. Histomorphometric quantification was performed in a blinded fashion by a trained pathologist, applying established histopathological scores of acute tubular damage. Two separate scores were used for histological evaluation of extent and severity of cortical tubular damage on whole image slides from both kidneys of each animal (Suppl. Table 1).^36^–^38^

### Statistical Analysis

Data are given as mean ± standard deviation unless otherwise specified. In normal distribution (according to the Kolmogorov–Smirnov), Student’s *t* test was used when comparing two groups, otherwise regular one-way analysis of variance (ANOVA) was applied. If distributional requirements were not met or variables were dichotomous, the nonparametric Mann–Whitney *U* test or *χ*^2^ test were used, respectively. Relationships between serum renal function parameters and histologically assessed tubular damage were studied using Pearson correlation. For univariate correlation analysis of dichotomous variables, the Phi coefficient and, for metric variables, the point-biserial correlation coefficient were used. Area under receiver operating characteristic curve (AUROCC) and binary multiple logistic regression analysis were performed using IBM SPSS software (version 22.0; SPSS Inc., Chicago, IL). Area under the curve (AUC) was computed using the trapezoidal rule, and an AUC > 0.7 was considered acceptable for diagnostic discrimination.^39^ Best cut-off values were calculated using the Youden-Index. *p* Values < 0.05 were considered significant, and *p* < 0.002 highly significant.

## Results

### Patient and Tumor Characteristics

Between 2007 and 2016, 153 patients were treated with HIPEC ± EPIC. Patient and tumor characteristics and perioperative/intraoperative details are presented in Table 3. There were slightly more female patients (*n* = 92, 58.4%; Suppl. Fig. 1A). Mean age was 58 ± 12.6 years, and mean body mass index (BMI) was 26 ± 5.6 kg/m^2^ (Suppl. Fig. 1B, C). Overall, 60 patients (38.2%) received neoadjuvant therapy prior to HIPEC treatment. Twelve different neoadjuvant regimens were used depending on the primary tumor: capecitabine (*n* = 4, 6.7%), cisplatin (*n* = 3, 5%), FLO (*n* = 2, 3.3%), FLOT (*n* = 10, 16.7%), FOLFOX(+Avastin) [*n* = 17 (4), 28.3% (6.7%)], FOLFIRI(+Avastin) [*n* = 2 (3), 3.3% (5%)], FOLFOXIRI + Avastin (*n* = 1, 1.7%), 5FU + LV (*n* = 3, 5%), and EOX (*n* = 4, 6.7%).

Patients spent 21.7 ± 13.1 days in hospital and 7.1 ± 7.4 days on ICU/IMC (4 ± 6.9 days when excluding patients with EPIC treatment) (Suppl. Fig. 1D). A majority of patients were treated with HIPEC due to PC of colorectal cancer and PMP of appendiceal origin [*n* = 52 (33.1%) and *n* = 47 (29.9%), respectively]. Twenty-seven patients (17.2%) underwent HIPEC for treatment of PC from gastric cancer, 14 patients (8.9%) because of mesothelioma, 12 patients (7.7%) because of PC from ovarian cancer, 4 patients (2.5%) because of PC from intestinal cancer, and 1 patient (0.6%) because of PC from signet-ring cell carcinoma of unknown primary (CUP).

Parietal peritoneum was completely or partially resected in 132 patients (86.3%). The remaining patients underwent peritonectomy prior to HIPEC surgery, or were already resected at external departments, and transferred to our department for HIPEC treatment only. Colon (segmental resection, hemicolectomy, subtotal or total colectomy, *n* = 86) and omentum (total omentectomy, *n* = 58) were the second and third most frequently resected organs, with pancreas (distal pancreatectomy) being the least frequently resected organ (*n* = 3) (Suppl. Table 2).

### HIPEC Regimens and Postoperative Complications

Overall, 157 HIPEC procedures were performed. Additional EPIC treatment was performed in 36 cases (23%). Four patients underwent HIPEC twice owing to local recurrence. PC from CRC was most frequently treated with bidirectional oxaliplatin intraperitoneally (i.p.), combined with leucovorin and 5-fluorouracil i.v. (*n* = 32, 62%) (Table 2). Treatment with combined doxorubicin and mitomycin C with or without 5-fluorouracil EPIC (*n* = 17, 33%) was abandoned in 2011. PMP of appendiceal origin was treated with doxorubicin and mitomycin C HIPEC (*n* = 29, 62%), or with doxorubicin and mitomycin C HIPEC in combination with 5-fluorouracil EPIC (*n* = 11, 23%). PC from gastric cancer was treated with doxorubicin and cisplatin until 2013 (*n* = 14, 52%), and afterwards with mitomycin C and cisplatin (*n* = 10, 37%). Patients with mesothelioma or PC from ovarian cancer underwent HIPEC with combined doxorubicin and cisplatin in 71% (*n* = 10) and 33% (*n* = 4) of cases, respectively. In PC from ovarian cancer, doxorubicin, and cisplatin HIPEC was more frequently combined with taxol EPIC (*n* = 8, 67%).

**Table 2:** HIPEC and EPIC regimens

	Primary	*N*
*HIPEC*		
DOX (15 mg/m^2^), CPL (50 mg/m^2^)	G, M, O, A	29
CPL (75 mg/m^2^)	M	1
CPL (75 mg/m^2^), MMC (30 mg/m^2^)	G, M	11
MMC (15 mg/m^2^)	CR, A	2
DOX (15 mg/m^2^), MMC (15 mg/m^2^)	CR, A	32
OXA (460 mg/m^2^), LV* (20 mg/m^2^), 5-FU* (400 mg/m^2^)	CR, A, I	39
OXA (460 mg/m^2^)	CR, A, G, I	5
OXA (460 mg/m^2^), MMC (15 mg/m^2^)	G	2
*HIPEC* + *EPIC*		
DOX (15 mg/m^2^), CPL (50 mg/m^2^) + TAX (80 mg/m^2^)	O, M	10
DOX (15 mg/m^2^), MMC (15 mg/m^2^) + 5-FU (650 mg/m^2^)	CR, A	25
OXA (460 mg/m^2^), LV* (20 mg/m^2^), 5-FU* (400 mg/m^2^) + 5-FU (650 mg/m^2^)	CR	1

*HIPEC* hyperthermic intraperitoneal chemotherapy, *EPIC* early postoperative intraperitoneal chemotherapy, *G* gastric cancer, *M* mesothelioma, *O* ovarian cancer, *A* appendiceal neoplasms, *CR* colorectal cancer, *I* intestinal cancer, *CPL* cisplatin, *MMC* mitomycin C, *DOX* doxorubicin, *TAX* taxol, *5-FU* 5-fluorouracil, *OXA* oxaliplatin, *LV* leucovorin

^*^i.v. chemotherapy (bidirectional)

The most frequent adverse event in the postoperative course was AKI (*n* = 50, 31.8%). Leucopenia, defined as WBCC < 3/nl, occurred in 28 patients (17.8%). Anastomotic leakage was the most frequent surgical complication (*n* = 11, 7%; predominantly affecting esophagojejunal and ileorectal anastomoses), followed by perforation (intestinal or bladder; *n* = 6, 3.8%), intraperitoneal abscess (*n* = 4, 2.5%), fistula (enteroatmospheric, enterocolic, vesicorectal, or gastropleural; *n* = 4, 2.5%), and pancreatic fistula (*n* = 3, 1.9%).

Thirty-day mortality was 1.3% (*n* = 2) (Table 3). In-hospital mortality was due to septic multiorgan failure in one case, and acute right ventricular failure due to fulminant pulmonary embolism in another.

**Table 3:** Patient and HIPEC characteristics

Parameter	*N* (%)
HIPEC mode	153 (97.5%)
HIPEC	121 (77%)
HIPEC + EPIC	36 (23%)
*Gender*	
Male	65 (41.4%)
Female	92 (58.6%)
Age (years)	58 ± 12.6
BMI (kg/m^2^)	26 ± 5.6
Neoadjuvant therapy	60 (38.2%)
Hospital time (days)	21.7 ± 13.1
ICU/IMC time (days)	7.1 ± 7.4
*Primary tumor*	
Colorectal	52 (33.1%)
Appendiceal	47 (29.9%)
Gastric	27 (17.2%)
Mesothelium	14 (8.9%)
Ovarian	12 (7.7%)
Intestine	4 (2.5%)
CUP	1 (0.6%)
*Postop. complications*	
Acute kidney injury	50 (31.8%)
Leucopenia	28 (17.8%)
Abscess	4 (2.5%)
Fistula*	4 (2.5%)
Pancreatic fistula	3 (1.9%)
Anastomotic leakage	11 (7%)
Perforation**	6 (3.8%)
30-Day mortality	2 (1.27%)

HIPEC, hyperthermic intraperitoneal chemotherapy, EPIC early postoperative intraperitoneal chemotherapy, *BMI* body mass index, *ICU* intensive care unit, *IMC* intermediate care unit, *CUP* carcinoma of unknown primary

^*^Enteroatmospheric, enterocolic, vesicorectal, gastropleural fistula

^**^Intestinal, bladder perforation

## Acute Kidney Injury in HIPEC Patients

AKI, defined according to KDIGO criteria, occurred in 35.5% of patients (*n* = 43) treated with HIPEC and in 19.4% of patients (*n* = 7) treated with HIPEC + EPIC. Demographic characteristics did not differ significantly in patients with and without renal injury (Table 4).

**Table 4:** Univariate analysis of risk factors for AKI after HIPEC

Parameter	AKI+	AKI−	*p* value	*r* value
*Gender*			0.07	
Male	26 (40%)	39 (60%)		
Female	24 (26%)	68 (74%)		
Age (years)	59 ± 12.8	56 ± 12.4	0.17	
BMI (kg/m^2^)	26.5 ± 6	25.4 ± 5.4	0.06	
Congestive heart failure	1 (20%)	4 (80%)	0.6	
Hypertension	16 (39%)	25 (61%)	0.16	
Chronic kidney disease	1 (33%)	2 (66%)	0.17	
Diabetes mellitus	2 (50%)	2 (50%)	0.39	
ACE inhibitor	3 (21.4%)	11 (78.6%)	0.44	
AT blocker	8 (53.3%)	7 (46.7%)	**0.04**	0.17^#^
Diuretics	6 (42.9%)	8 (57.1%)	0.29	
Hospital time (days)	22.9 ± 13.3	21.3 ± 13	0.38	
ICU/IMC time (days)	9.1 ± 12.1	5.2 ± 4.2	0.12	
Neoadjuvant CTx	23 (38.3%)	37 (61.7%)	0.25	
Cisplatin neoadjuvant	1 (33%)	2 (66%)	1	
IO transfusion	10 (20.4%)	39 (79.6%)	0.09	
IO vasopressors	40 (29.5%)	102 (70.5%)	**0.02**	− 0.19^#^
IO fluid (ml/kg/bw)	74.1 ± 38.2	71.4 ± 42.6	0.85	
IO SBP < 100 mmHg (min)	37.5 ± 40.3	30 ± 32.8	0.28	
Cisplatin HIPEC	31 (62%)	19 (38%)	**<** **0.001**	0.49^#^
Preoperative creatinine	0.81 ± 0.2	0.73 ± 0.2	**0.01**	0.21^##^
Preoperative urea	28.1 ± 8.5	24 ± 8.1	**0.005**	0.23^##^
*Primary tumor*			**< 0.001**	− 0.49^#^
Colorectal	11 (21.2%)	41 (78.8%)		
Appendiceal	5 (10.6%)	42 (89.4%)		
Gastric	17 (63%)	10 (37%)		
Mesothelium	9 (64.3%)	5 (35.7%)		
Ovarian	7 (58.3%)	5 (41.7%)		

*HIPEC* hyperthermic intraperitoneal chemotherapy, *AKI* acute kidney injury, *BMI* body mass index, *ACE* angiotensin-converting-enzyme, *AT* angiotensin, *ICU* intensive care unit, *IO* intraoperative, *IMC* intermediate care unit, *CTx* chemotherapy, *SBP* systolic blood pressure, *CUP* carcinoma of unknown primary, *bw* body weight

^**#**^According to Phi-coefficient/Cramers V

^##^According to point-biserial correlation

Boldface indicates *p* < 0.05

A majority of patients undergoing HIPEC for treatment of mesothelioma and PC from gastric or ovarian cancer developed AKI (64.3%, 63%, and 58.3%, respectively), whereas AKI occurred less frequently in patients treated for PC from colorectal cancer or PMP of appendiceal origin (21.2% and 10.6%, respectively; *p* < 0.001).

For further analysis of HIPEC-induced AKI, patients with urinary tract obstruction (*n* = 1) or AKI occurring concomitantly with septic complications (*n* = 4) were excluded.

**Fig. 1:**
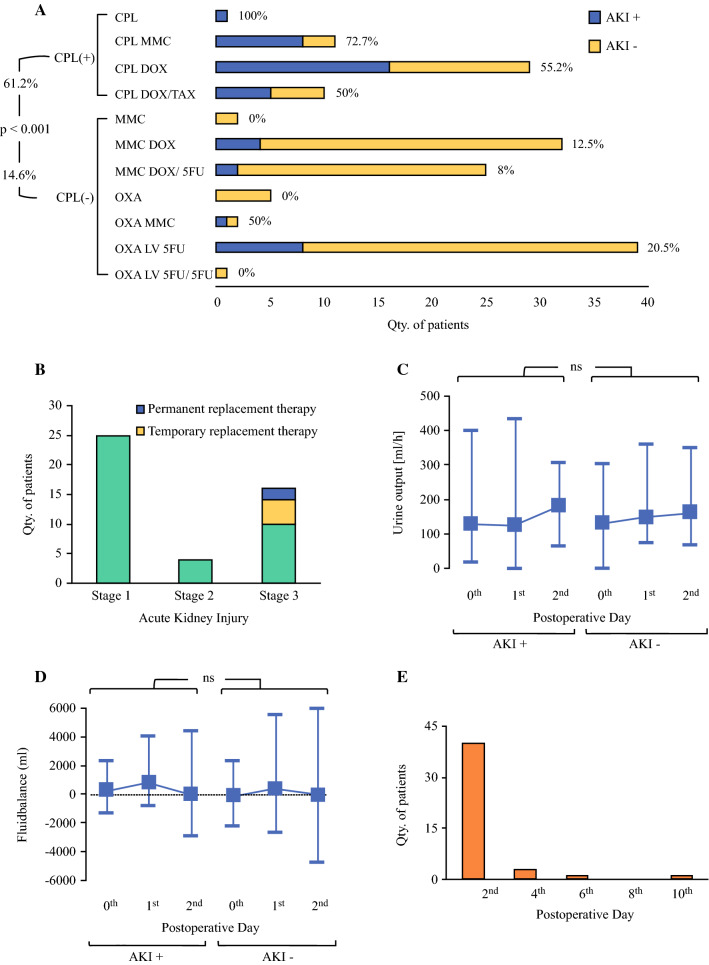
HIPEC-induced acute kidney injury in patients. **a** Occurrence of AKI after HIPEC regimens containing (CPL+) and not containing (CPL−) cisplatin. *CPL* cisplatin, *DOX* doxorubicin, *LV* leucovorin, *MMC* mitomycin C, *OXA* oxaliplatin, *TAX* taxol, *5-FU* 5-fluorouracil. **b** Staging of patients (*n* = 45) with HIPEC-induced kidney injuries according to KDIGO classification. **c** Comparison of urine output and **d** fluid balance between patients with (AKI+) and without (AKI−) kidney injury after HIPEC on day of operation (0th), 1st, and 2nd POD. Data are expressed as mean ± range. **e** Quantity of patients diagnosed with HIPEC-induced AKI per postoperative day. *p < 0.05; ^ns^*p* ≥ 0.05

In total, 61.2% (*n* = 30) of patients treated with cisplatin-containing HIPEC regimens but only 14.6% (*n* = 15) of those treated with cisplatin-free regimens developed AKI (*p* < 0.001) (Fig. 1a).

According to KDIGO classification, 55.6% of patients with HIPEC-induced AKI (*n* = 25) suffered stage 1, 8.8% (*n* = 4) stage 2, and 35.6% (*n* = 16) stage 3 renal injury (Fig. 1b). Six patients with HIPEC-induced stage 3 AKI required renal replacement therapy, four of those temporarily and two permanently. All patients requiring hemodialysis were treated with cisplatin-containing HIPEC. Univariate analysis confirmed cisplatin-containing HIPEC as a risk factor for severe stage 3 renal injury (*p* = 0.004; *r* = 0.427). Only one of the patients with stage 3 renal injury was not treated with cisplatin. Furthermore, female patients were at risk for stage 3 injury (*n* = 11 versus *n* = 5 male patients, respectively; *p* = 0.048; *r* = 0.295). Patients with HIPEC-induced AKI had significantly higher preoperative serum creatinine and urea values compared with those without HIPEC-induced AKI (*p* = 0.009; Suppl. Fig. 2A, B). However, receiver operating characteristic (ROC) and AUC analyses revealed insufficient potential of preoperative creatinine and urea to discriminate between patients with and without AKI (Suppl. Fig. 3A, B). Intraoperative use of vasopressor agents inversely correlated with renal injury (r = − 0.19), but intraoperative hypotension (defined as episodes of systolic blood pressure < 100 mmHg) and fluid influx did not differ between patients with and without AKI. Patients receiving vasopressors had significantly longer episodes of intraoperative hypotension (30 ± 36 versus 10 ± 17.5 min; *p* = 0.01) and higher fluid influx (76.3 ± 40.1 versus 47.8 ± 23.2 ml/kg body weight; *p* = 0.006). Serum inflammatory markers WBCC and CRP, and liver synthesis markers albumin and Quick’s value, were comparable in patients with and without AKI (Suppl. Fig. 2C–F). LDH levels were significantly increased in AKI+ patients on POD 4, 6, and 8 (Suppl. Fig. 2G).

Urine output did not differ significantly between AKI+ and AKI− patients (Fig. 1c), and fluid balance was likewise comparable in both groups (Fig. 1d).

AKI was diagnosed by an increase of serum creatinine in 40 patients (89%) on POD 2, in three patients on POD 4, and in one patient each on POD 6 and POD 10 (Fig. 1e). Two patients also met the KDIGO criterion of AKI of urine output less than 0.5 ml/kg/h for 6 h on POD 2.

Univariate analysis of risk factors (Table 4) revealed strongest correlations between cisplatin-containing HIPEC and AKI (*r* = 0.49). Increased preoperative urea (*r* = 0.23) and creatinine levels (*r* = 0.21), and angiotensin receptor blockers (ARB) (*r* = 0.17) likewise correlated with AKI. Notably, administration of angiotensin-converting enzyme (ACE) inhibitors and ARB was paused on the day of surgery and HIPEC. Multiple logistic regression analysis confirmed cisplatin-containing regimens as an independent predictive risk factor for AKI after HIPEC (Table 5).

**Table 5:** Binary multiple logistic regression of risk factors for AKI after HIPEC

Variable	OR	95% CI of OR	*p*-Value
Cisplatin*	16.299	6.225–42.676	< 0.001
Preoperative creatinine	8.198	0.732–91.823	0.088
Preoperative urea	1.044	0.982–1.11	0.164
Angiotensin receptor blocker	7.598	2.036–28.356	0.003

*AKI* acute kidney injury, *HIPEC* hyperthermic intraperitoneal chemotherapy, *OR* odds ratio, *CI* confidence interval

^*^Cisplatin-containing HIPEC regimen

### HIPEC-Induced Acute Kidney Injury in a Preclinical Mouse Model

**Fig. 2:**
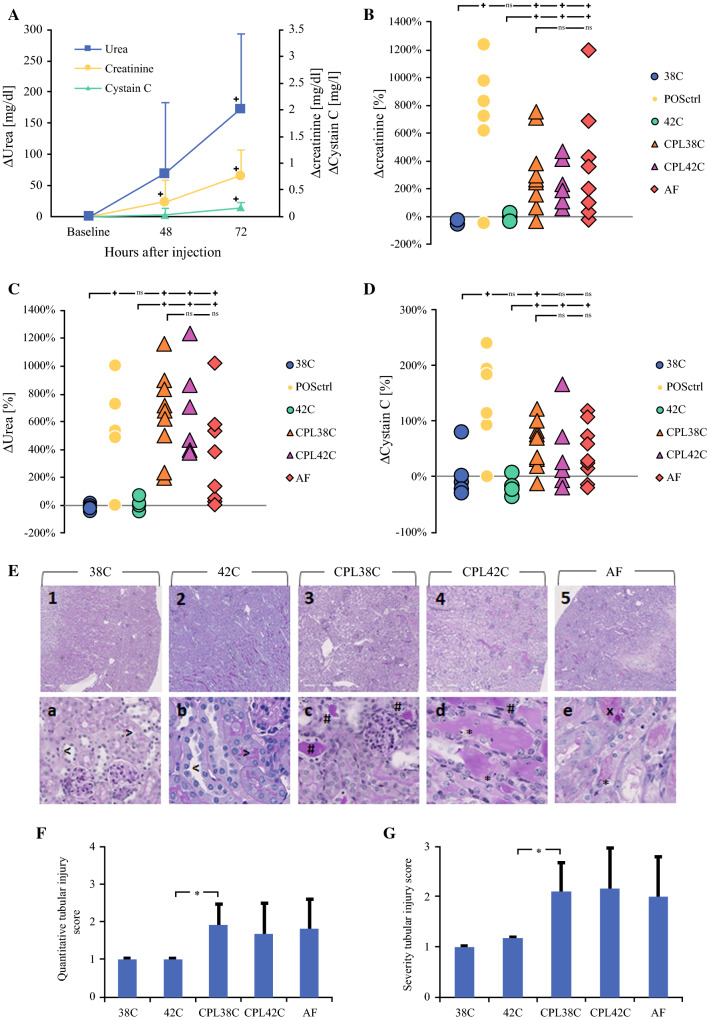
Influence of cisplatin, hyperthermia, and amifostine on HIPEC-induced AKI in a preclinical mouse model. **a** Plasma levels of creatinine, urea, and cystatin C, 48 h and 72 h after single i.p. injection of cisplatin (20 mg/kg). Data are expressed as mean ± SD; *n* = 6, *p < 0.05. **b**–**d** Percentage changes in creatinine (**b**), urea (**c**) and cystatin C levels (**d**), 72 h after i.p. perfusion (90 min) with saline (0.9%; circles) or cisplatin (75 mg/m^2^ in 2 L/m^2^ perfusate; triangles), or after single i.p. injection with cisplatin as a positive control (20 mg/kg; black circles, POSctrl). Perfusion was performed under either normothermia (38 °C, open circles, 38C; and open triangles, CPL38C) or under hyperthermic conditions (41–42 °C; gray circles, 42C; and gray triangles, CPL42C). Amifostine (200 mg/kg; diamonds, AF) was injected s.c. 15 min prior to perfusion with cisplatin at 38 °C. Individual animals are expressed as one datapoint each. *n* = 6 (38C, 42C, CPL42), *n* = 10 (CPL38, AF); *p < 0.05; ^ns^*p* ≥ 0.05. **e** Representative PAS staining revealing histopathological changes in murine kidneys, 72 h after i.p. perfusion with saline at 38 °C (left panels, 38C), saline at 42 °C (middle left panels, 42C), cisplatin (75 mg/m^2^) at 38 °C (center panels, CPL38C), cisplatin at 42 °C (middle right panels, CPL42C), or cisplatin at 38 °C after amifostine treatment (200 mg/kg s.c.; right panels, AF). Note hyaline casts (*#*), apoptosis (*), and necrosis (*x*) of tubular epithelial cells in kidneys of animals perfused with cisplatin (in center and right panels), and regular proximal (>) and distal (<) tubules in kidneys of those treated with saline (left panels). Images in upper panels are taken at 100× magnification and in lower panels at 400× magnification. **f**, **g** Histomorphometric scoring of tubular injury quantity (**f**) and severity (**g**), 72 h after i.p. perfusion with saline at 38 °C (38C), saline at 42 °C (42C), cisplatin at 38 °C (CPL38C), cisplatin at 42 °C (CPL42C), or cisplatin at 38 °C after amifostine treatment (AF). Data are expressed as mean ± SD, *p < 0.05

An experimental mouse model was applied to unravel the significance of cisplatin versus hyperthermia in HIPEC-induced AKI, and putative preventive effects of the cytoprotective agent amifostine. As a positive control and to determine optimal timing of blood withdrawal for further analyses, renal function parameters (creatinine, urea, cystatin C) were measured 48 h and 72 h after i.p. injection of a single cisplatin dose (20 mg/kg) (Fig. 2a). Forty-eight hours after injection, plasma levels of creatinine and urea, but not cystatin C, were elevated compared with baseline levels. Seventy-two hours after i.p. cisplatin exposure, levels of creatinine, urea, and cystatin C were consistently and significantly increased compared with baseline levels (*p* = 0.05, *p* = 0.02, and *p* = 0.04, respectively). In subsequent experiments, HIPEC-induced AKI was therefore quantified 72 h following HIPEC.

To determine the selective contribution of hyperthermic perfusion and cisplatin HIPEC to AKI, mice were treated by i.p perfusion with saline (0.9%) or cisplatin (75 mg/m^2^ in saline 0.9%) at normothermia (38 °C) or hyperthermia (42 °C). Baseline plasma levels of creatinine, urea and cystatin C were determined seven days prior to i.p. perfusion. Seventy-two hours after perfusion with cisplatin, plasma levels of creatinine, urea, and cystatin C (triangle symbols in Fig. 2b–d, respectively) were highly and significantly elevated compared with baseline levels, indicating kidney dysfunction. Remarkably, however, these changes occurred to a similar extent upon cisplatin perfusion at normo- and hyperthermia (open and gray triangle symbols, respectively, in Fig. 2b–d). By contrast, perfusion with saline at normo- or hyperthermia (open and gray circle symbols, respectively, in Fig. 2b–d) did not cause any increase in plasma levels of creatinine, urea, or cystatin C. Intraperitoneal injection of a single cisplatin dose (20 mg/kg) was applied as a positive control, and expectedly caused a stark increase in plasma levels of all three renal function markers (black circle symbols in Fig. 2b–d).

Consistent with these findings, microscopic analysis of PAS-stained kidney sections revealed extensive signs of tubular damage in mice perfused with cisplatin at both normothermia (CPL38C; center panels in Fig. 2e) or hyperthermia (CPL42C; middle right panels in Fig. 2e). Notably, such histological signs of kidney damage were mostly absent in mice perfused with saline at normothermia (38C; left panels in Fig. 2e) or hyperthermia (42C; middle left panels in Fig. 2e). Histomorphometric scoring was performed applying quantitative and semiquantitative scores (Suppl. Table 1), and confirmed tubular damage of comparable quantity (Fig. 2g) and severity (Fig. 2f) in mice treated with CPL38C and CPL42C, whereas only mild tubular damage was observed in animals treated with 38 °C and 42 °C.

Finally, we aimed to assess the potential of the cytoprotective agent amifostine to prevent HIPEC-associated AKI. Amifostine (200 mg/kg s.c.) was administered immediately prior to i.p. perfusion with cisplatin at 38 °C. However, pretreatment with amifostine (AF) did not cause any reduction of renal function parameters (Fig. 2b–d) or tubular damage (Fig. 2e–g) compared with i.p. perfusion with cisplatin in the absence of amifostine (CPL38C).

Taken together, these results from a preclinical mouse model confirm our clinical observation that nephrotoxic effects of cisplatin are a chief reason underlying HIPEC-induced AKI and indicate that the effects of hyperthermic intraperitoneal perfusion alone seem to be negligible in this context.

## Discussion

Peritoneal metastases are accompanied with poor outcome.^6^,^11^–^13^ Currently, cytoreductive surgery (CRS) and HIPEC offer best outcomes for selected patients, depending on primary disease.^14^,^40^ However, HIPEC is associated with relevant morbidity.^41^–^43^ Several patients develop postoperative AKI, which increases overall morbidity and mortality.^23^,^24^,^27^ Identifying pathogenetic factors through which HIPEC causes AKI is crucial for the development of strategies to reduce HIPEC-related morbidity. In this study, we retrospectively analyzed the prevalence and risk factors of AKI in patients treated with HIPEC. Furthermore, we evaluated the relevance of hyperthermic intraperitoneal perfusion and cisplatin as pathogenic factors underlying HIPEC-induced AKI. Based on our findings, we identify cisplatin as the main risk factor for HIPEC-induced AKI, whereas hyperthermia plays an insignificant role in this context.

The reported prevalence of AKI after HIPEC displays vast variability, ranging from 0.9 to 48%.^27^,^44^ Systemic treatment with cisplatin leads to AKI in 20% of patients, prompting the assumption that patients treated with cisplatin-containing HIPEC are at comparable risk.^45^,^46^ Indeed, literature suggests that cisplatin-containing HIPEC regimens are frequently accompanied with AKI (Table 1), and cisplatin has been identified as an independent risk factor for AKI in different cohorts of HIPEC patients.^18^,^26^ Several other clinical studies, however, failed to identify cisplatin as an independent risk factor for HIPEC-induced AKI.^19^,^23^,^24^ Instead, several alternative factors, in particular hyperthermia and surgery-related cytokine release, have been supposed to induce prerenal AKI due to vasodilatation of splanchnic vessels, resulting in relative hypovolemia.^30^ In our patient collective, cisplatin was identified as the main risk factor for AKI after HIPEC. All patients requiring renal replacement therapy had been treated with cisplatin-containing regimens. Obviously, other drugs may have additionally contributed to HIPEC-induced AKI. Indeed, a recent systematic review reported an incidence of 13.4% of postoperative AKI after major abdominal surgeries.^47^

In our cohort, patients medicated with ARB were at an increased risk for HIPEC-induced AKI, which is in accordance with the findings of Hakeam et al.^20^ ARB are potentially nephrotoxic in acute settings but nephroprotective in the long term.^48^ In preclinical models, both protective and aggravating effects of ARB in cisplatin-derived kidney injury have been reported, but underlying mechanisms remained elusive^49^,^50^.

Consistent with our present findings, Sin et al. identified baseline creatinine levels as an independent risk factor for HIPEC-associated AKI.^23^ Concerning associations with primary tumors, postoperative AKI was frequently observed in patients suffering mesothelioma and PC from gastric or ovarian cancer. We consider this attributable to the frequent use of cisplatin-containing HIPEC regimens in these tumor entities. Somewhat surprisingly, patients receiving vasopressors intraoperatively developed AKI less frequently. Though norepinephrine constricts renal arterioles and reduces renal blood flow, it is considered safe in the setting of AKI.^51^ Both, intraoperative duration of hypotension and fluid influx, were significantly higher in patients treated with vasopressors, suggesting that nephroprotective features of vasopressors are not mediated by enhanced renal perfusion due to elevated systemic blood pressure. Hypotension and fluid influx did not correlate with HIPEC-induced AKI, supporting the hypothesis of primarily chemotoxicity-provoked renal injury.

HIPEC-associated AKI can trigger further complications and enhance overall morbidity.^24^ Identifying patients at risk would, therefore, facilitate postoperative management. In our population, 89% of patients with AKI were diagnosed by an increase in creatinine values on POD2.^35^ It thus appears reasonable to recommend postoperative observation of HIPEC patients on ICU/IMC until POD2, especially when any of the above-mentioned risk factors apply. Multiple recent studies found goal-directed fluid management strategies, guided by hemodynamic parameters, to be advantageous over liberal fluid management strategies in reducing perioperative morbidity of patients treated with CRS and HIPEC.^52^,^53^ Whether preoperative hydration strategies are of additional value has not been substantiated with clinical studies yet.

To determine whether hyperthermia, cisplatin, or both predispose to HIPEC-induced AKI,^54^,^55^ we applied a model of experimental HIPEC in mice. Consistent with the presented clinical data, renal injury was highly prevalent after perfusion with cisplatin in this preclinical model, whereas hyperthermic perfusion did not induce or enhance renal injury when administered alone or in combination with cisplatin, respectively. It would obviously have been interesting to compare nephrotoxic effects induced by various different HIPEC regimens applying this preclinical model. However, mice are highly intolerant to alternative HIPEC regimens such as oxaliplatin and mitomycin c, thus precluding such further analyses.^31^ We likewise applied the preclinical HIPEC model to assess putative benefits of amifostine, a cytoprotective adjuvant applied in chemotherapy, in alleviating HIPEC-associated nephrotoxicity. Although renal injury appeared to be slightly less severe in mice treated with amifostine prior to perfusion with cisplatin, this putative nephroprotective effect did not reach statistical significance. However, interpretation of renal injury by alteration of serum renal function parameters in mice remains technically challenging^46^,^56^, and we thus do not intend to rule out a nephroprotective effect of amifostine in prophylactic treatment of AKI induced by cisplatin-containing HIPEC. Further in-depth (pre-)clinical studies are undoubtedly necessary to better understand the pathogenesis of HIPEC-induced AKI, and to evaluate the potential of additional nephroprotective agents. It would, in this context, be a reasonable effort to modify the described preclinical model for application in other species to facilitate further pharmacokinetic investigation in this direction.

Remarkably, clinical evidence for strategies to prevent cisplatin-induced AKI is scarce. Van Driel et al., who did not observe significant nephrotoxicity in a collective of 118 patients treated with cisplatin HIPEC, routinely applied administered sodium thiosulphate (9 g/m^2^) to prevent nephrotoxicity. However, true nephroprotective effects of this approach cannot be inferred from this study since a specific control group is missing, and since fractionated application of the cisplatin dose may likewise have contributed to nephroprotection.^14^ Based on a retrospective study including 52 patients, Bouhadjari et al. reported a 13% prevalence of severe renal impairment in patients undergoing cisplatin HIPEC with preinterventional application of amifostine (910 mg/m^2^) as compared with a 33% prevalence in patients undergoing cisplatin HIPEC without amifostine; however, postinterventional changes in mean creatinine clearance did not differ between both groups.^22^ Taken together, recommendations concerning specific nephroprotective drugs and strategies cannot be given based on current clinical evidence.

## Conclusions

Our present study implies that the prevalence of AKI in patients undergoing HIPEC is high, and predominantly caused by cytotoxic side effects of chemotherapeutic drugs. Application of cisplatin-containing HIPEC regimens is an independent risk factor of HIPEC-induced AKI. Cisplatin-containing HIPEC regimens should therefore be applied with caution and restricted to selected indications where cisplatin HIPEC has proven oncological benefits over alternative drugs. Further independent risk factors for HIPEC-induced AKI are angiotensin receptor blockers and preoperative creatinine and urea levels. Preclinical data suggest that hyperthermic perfusion of the abdomen by itself plays a minor causative role in HIPEC-induced AKI, and that HIPEC-induced AKI cannot be prevented by application of the cytoprotective agent amifostine.

## Supplementary Information

Below is the link to the electronic supplementary material.

Supplemental Figure 1 (A) Gender, (B) age, (C) BMI and (D) hospitalization time with time spent on ICU/IMC of patients with (AKI+) and without kidney injury (AKI-) after HIPEC. Data are expressed as mean ± range. *p < 0.05; ^ns^*p* ≥ 0.05 (TIF 939 KB)

Supplemental Figure 2 (A) Creatinine, (B) Urea, (C) WBCC, (D) CRP, (E) Albumin, (F) quick and (G) LDH values of patients with (AKI+) and without (AKI-) kidney injury after HIPEC every other day from day of operation (0th) until 10th POD. Data are expressed as mean ± range. *p < 0.05; **p < 0.002; ^ns^*p* ≥ 0.05 (TIF 18926 KB)

Supplemental Figure 3 Receiver operating characteristic (ROC) curve for preoperative creatinine (A) and preoperative urea (B) levels for prediction of acute kidney injury (AKI) after HIPEC. AUC for both, creatinine (p=0.009) and urea (p=0.013), is 0.63. Best cut-off value for creatinine is 0.69mg/dl (sensitivity 78%, specificity 50%, NPV 85.5%, PPV 40%) and for urea 25.5mg/dl (sensitivity 63%, specificity 59%, NPV 79.7%, PPV 39.7%) (TIF 606 KB)

Supplementary file 4 (DOCX 13 KB)

Supplementary file 5 (DOCX 15 KB)
