# Supplementary figures and images for: HIPEC-Induced Acute Kidney Injury: A Retrospective Clinical Study and Preclinical Model
Source: Ann Surg Oncol. 2021 Jul 14;29(1):139–51. doi: 10.1245/s10434-021-10376-5 (PMC8677640; doi:10.1245/s10434-021-10376-5)

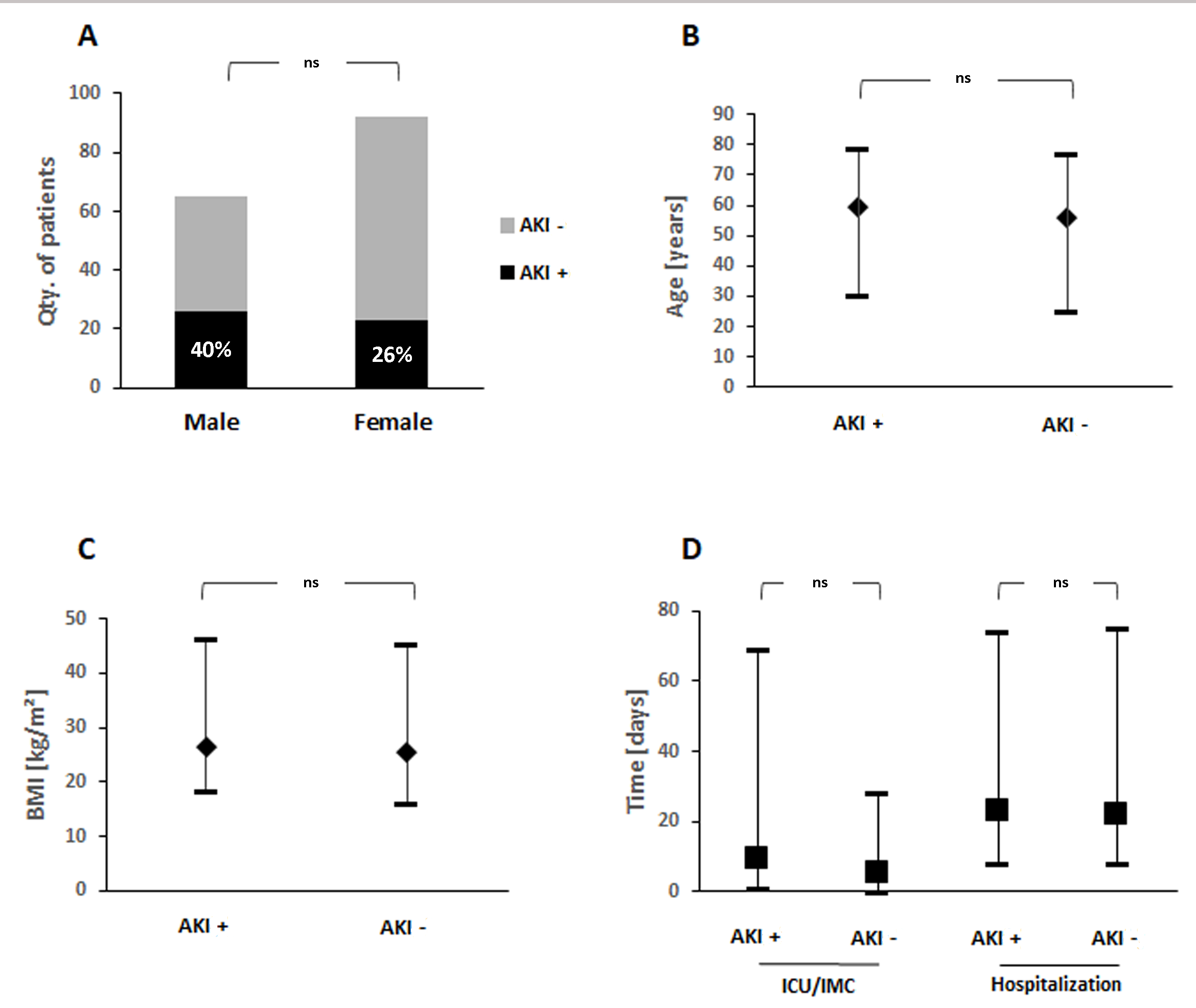

Supplement: Supplementary file 1 — (A) Gender, (B) age, (C) BMI and (D) hospitalization time with time spent on ICU/IMC of patients with (AKI+) and without kidney injury (AKI-) after HIPEC. Data are expressed as mean ± range. *p < 0.05; nsp ≥ 0.05 (TIF 939 KB) [file 10434_2021_10376_MOESM1_ESM.tif]

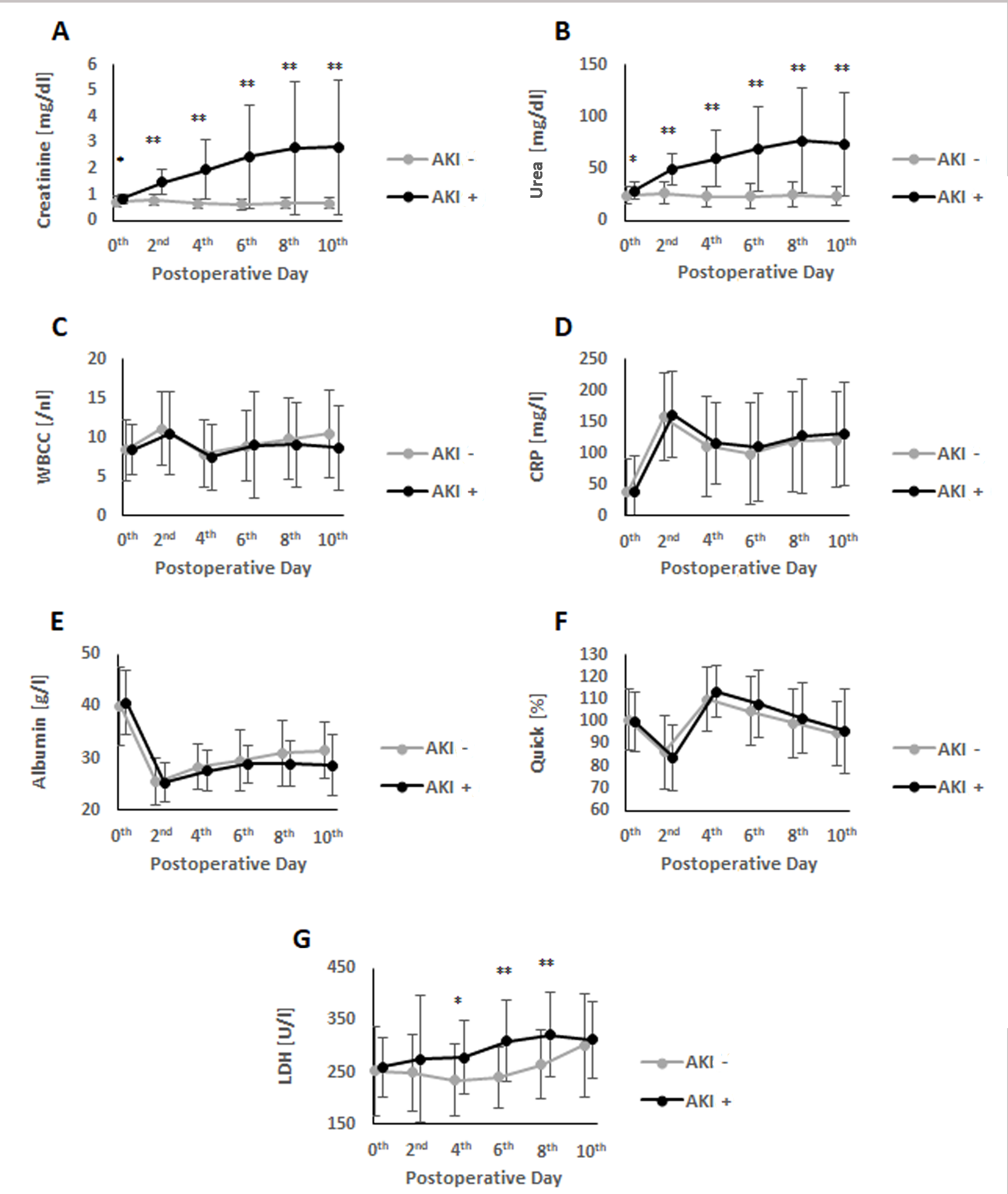

Supplement: Supplementary file 2 — (A) Creatinine, (B) Urea, (C) WBCC, (D) CRP, (E) Albumin, (F) quick and (G) LDH values of patients with (AKI+) and without (AKI-) kidney injury after HIPEC every other day from day of operation (0th) until 10th POD. Data are expressed as mean ± range. *p < 0.05; **p < 0.002; nsp ≥ 0.05 (TIF 18926 KB) [file 10434_2021_10376_MOESM2_ESM.tif]

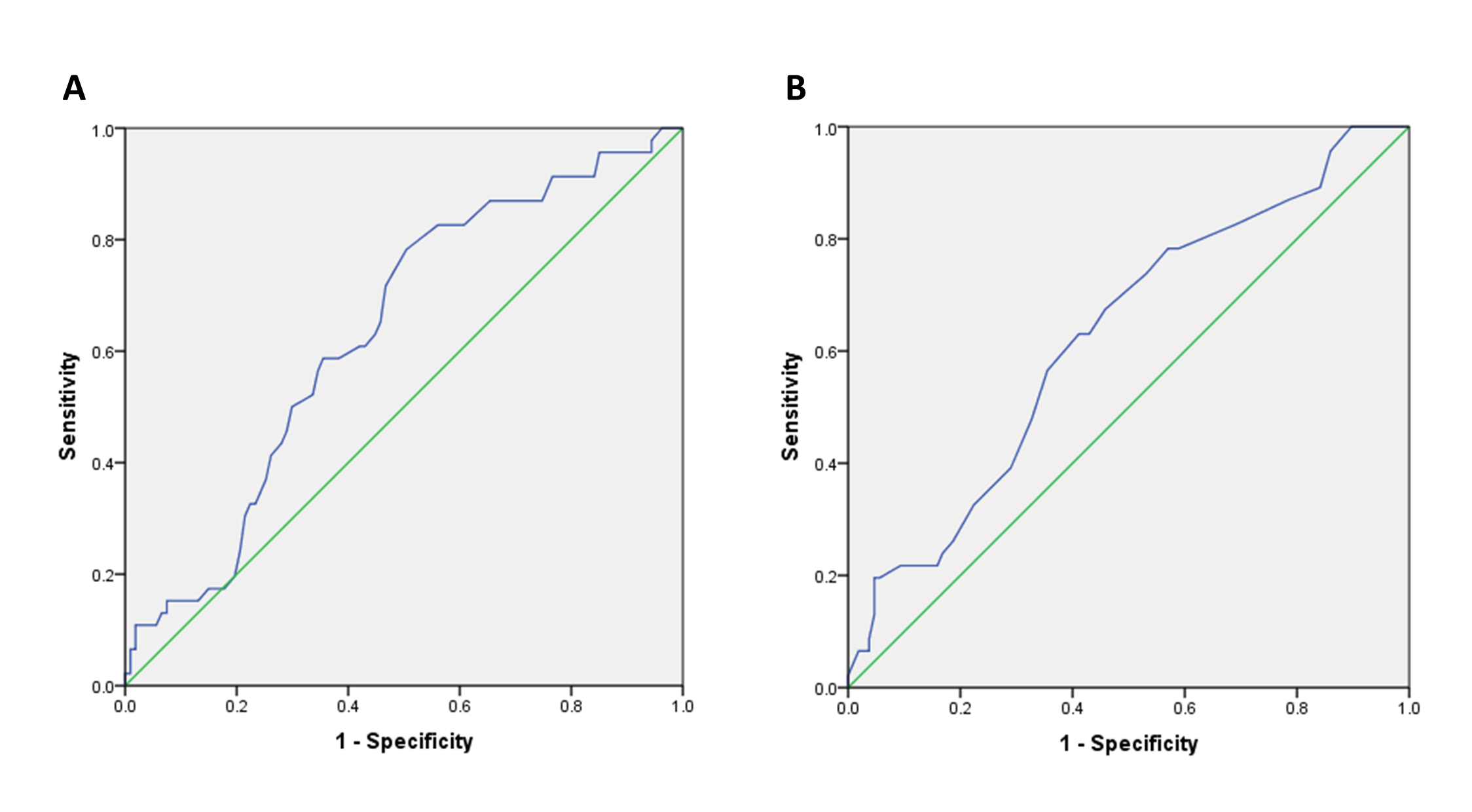

Supplement: Supplementary file 3 — Receiver operating characteristic (ROC) curve for preoperative creatinine (A) and preoperative urea (B) levels for prediction of acute kidney injury (AKI) after HIPEC. AUC for both, creatinine (p=0.009) and urea (p=0.013), is 0.63. Best cut-off value for creatinine is 0.69mg/dl (sensitivity 78%, specificity 50%, NPV 85.5%, PPV 40%) and for urea 25.5mg/dl (sensitivity 63%, specificity 59%, NPV 79.7%, PPV 39.7%) (TIF 606 KB) [file 10434_2021_10376_MOESM3_ESM.tif]
