# Supplementary material for: HIPEC-Induced Acute Kidney Injury: A Retrospective Clinical Study and Preclinical Model
Source: Ann Surg Oncol. 2021 Jul 14;29(1):139–51. doi: 10.1245/s10434-021-10376-5 (PMC8677640; doi:10.1245/s10434-021-10376-5)
Supplement: Supplementary file 4 — (DOCX 13 KB) [file 10434_2021_10376_MOESM4_ESM.docx]

**Supplemental Table 1: Histological quantitative and semiquantitative scores for assessment of tubular damage.**

| Points | Quantitative (% of damaged tubules) | Semiquantitative (severity) |
| --- | --- | --- |
| 1 | 0-25% | No to discrete tubular damage |
| 2 | 25-50% | Loss of brush border, luminal dilatation, hyaline cylinders |
| 3 | 50-75% | Epithelial atrophy, tubular necrosis, extended protein precipitation |
| 4 | 75-100% | n/a |
