# Supplementary material for: HIPEC-Induced Acute Kidney Injury: A Retrospective Clinical Study and Preclinical Model
Source: Ann Surg Oncol. 2021 Jul 14;29(1):139–51. doi: 10.1245/s10434-021-10376-5 (PMC8677640; doi:10.1245/s10434-021-10376-5)
Supplement: Supplementary file 5 — (DOCX 15 KB) [file 10434_2021_10376_MOESM5_ESM.docx]

**Supplemental Table 2: Frequency of organ resections.**

| Organ resected | N (%) | AKI+ | AKI- |
| --- | --- | --- | --- |
| Colon | 86 (54.8%) | 17 (19.8%) | 69 (80.2%) |
| Rectum | 40 (25.5%) | 10 (25%) | 30 (75%) |
| Intestine | 31 (19.7%) | 9 (29%) | 22 (71%) |
| Uterus | 18 (11.5%) | 2 (11.1%) | 16 (88.9%) |
| Ovary | 29 (18.5%) | 5 (17.2%) | 24 (82.8%) |
| Peritoneum | 132 (84.1%) | 39 (29.5%) | 93 (70.5%) |
| Stomach | 26 (16.6%) | 11 (42.3%) | 15 (57.7%) |
| Spleen | 30 (19.1%) | 6 (20%) | 24 (80%) |
| Omentum | 58 (36.9%) | 10 (17.2%) | 48 (82.8%) |
| Liver | 25 (15.9%) | 5 (20%) | 20 (80%) |
| Pancreas | 3 (1.9%) | 2 (4.4%) | 1 (0.9%) |

AKI: Acute Kidney Injury (+with/-without)
